# Some Clinical Aspects of Sterility
*Based upon a short paper read before the Bath and Bristol Branch of the British Medical Association on Oct. 27th, 1926.


**Published:** 1927

**Authors:** R. S. S. Statham

**Affiliations:** Honorary Gynæcologist, Bristol Royal Infirmary


### SOME CLINICAL ASPECTS OF STERILITY.*

BY

R. S. S. Statham, O.B.E., M.D., Ch.M.,

*Honorary Gynæcologist, Bristol Royal Infirmary.*

Sterility is commoner than is generally known, and gives rise to much distress, especially amongst the industrial class of patients. I am often told at the Royal Infirmary "my husband will leave me if he cannot get a child from me," and the patient suffers from a sense of inferiority almost equal to that which prevails in the East.

The usual classification adopted is that of absolute and relative sterility, and the causes are protean, but can be roughly divided into :—

(1) Those consisting of some anatomical defect which will entirely prevent fertilisation or ovulation.

(2) Those following upon some pathological impairment of a previously healthy genital tract.

(3) Those dependent on defects in the husband.

It is obvious that careful investigation and diagnosis are of paramount importance. The husband should never be forgotten in the investigation, and this is well illustrated by a case sent to me in 1923.

The patient had been married five years and had never become pregnant. There was nothing in her history or physical examination to account for her sterility, and I could obtain no account of any difficulty or abnormality in the sexual relations.

* Based upon a short paper read before the Bath and Bristol Branch of the British Medical Association on Oct. 27th, 1926.

I interviewed the husband, who confirmed this, but finally told me he had been operated on when quite young for "hernia." I found him to be a double cryptorchid. A specimen of seminal fluid was reported as being quite normal except for complete azoospermia. The man had no idea that he was in any way abnormal sexually, as he believed that the placing of his testicles within the abdomen was a usual procedure in cure of hernia.

One of the most striking features of the cases I have seen is the regularity with which they have been treated by dilating and curetting, many of the patients having undergone this operation more than once.

Now, when sterility is due to infantile uterus, a fairly common condition always accompanied by intrinsic (spasmodic) dysmenorrhœa, this procedure is of great value. My personal experience, however, is that by far the commonest causes are: first, endocrine upset usually associated with obesity; second, old pelvic peritonitis leading to pathological conditions of tubes and ovaries, and it is with these two conditions that this short paper deals. In parenthesis, it is obvious that dilating the cervix is mere waste of the patient's time and money if either of the above conditions exists.

*Endocrine Sterility.*—This condition appears to be very common locally, and is associated with obesity and a varying degree of hypothyroidism. Presumably the local limestone formation is a predisposing cause.

The thyroid and the ovary are interdependent to a very large degree, and administration of thyroid gland substance has a very stimulating action on ovarian activity. I have been unable to obtain any action whatever from "ovarian extract." I have given 50 grs. in one dose and found no toxic symptoms develop. I cannot think that a drug which is not toxic in such quantity can exert any therapeutic action in the usual doses of $\frac{1}{2}$—3 grains.

The victim of endocrine sterility is almost always fat. She gives the history of having rapidly put on flesh for some years or months and, at the same time, of her periods becoming more and more scanty. Often she will state that they last but a few hours. She is slow mentally, and complains of her hair falling out, especially from the outer half of the eyebrow. This type of patient may put on weight to an enormous extent. One of my cases weighed 9 stone at marriage, and when seen by me four years later weighed just over 18 stone!

The treatment I adopt is the administration of thyroid gland in large and increasing doses. In my early cases I admitted the patient to the wards so as to keep them under observation, but I found it was quite unnecessary.* I start with a daily dose of 5 grs. thyroid (=1 gr. sicca) and add 2 grs. each week. The patient should be seen weekly and weighed. She is instructed to complain at once of any palpitations or "dizzy attacks." In most cases no effect is observed until the dose is in the region of gr. 15 a day. I continue increasing the dose until symptoms of hyperthyroidism are produced, and I find that this occurs, as a rule, at between 20 and 30 grs. a day. When this stage is reached the dose is halved and the patient watched to see if that dose will suffice, or if it can be lowered still more. Most of my cases are now on a permanent dose of from 3 to 10 grs. a day, with "rest periods" of a couple of months at intervals.

The effects of this treatment are usually dramatic.

* Note.—Thyroid gland should always be ordered with its equivalent in dry gland stated; *e.g.* Ext. Thyroid gr. 10 (=2 gr. sicca), for some chemists dispense *whole* gland and some dry gland. One of my patients on 20 grs. a day changed her chemist, and this produced symptoms of severe toxicity in a few days. I found she was taking 20 grs. "sicca," equivalent to 100 grs. whole gland a day.

The patient loses weight rapidly, often 5 or 6 lbs. a week. The periods become normal. The mentality improves, and in a number of cases pregnancy has occurred within twelve months of the commencement of the treatment. As all these patients who conceived had been married over three years, I think it is a fair inference that the thyroid was the direct curative agent, especially as the menstrual periods had become normal in each case. I quote one illustrative case.

Mrs. A. B. came to the Royal Infirmary complaining of sterility and great increase of weight. Married five years, weight 17 st. 5 lb., "used to weigh about 8 stone." Her period came on three or four times a year, and "was only a few spots." She used to menstruate for six or seven days. She was put on increasing doses of thyroid, and at the end of seven months her weight was 12 st. 2 lb.; her periods were regular and she saw them for four days. She was then taking 25 grs. a day (=5 grs. sicca). She became pregnant after nine months' treatment, and gave birth to a daughter in the Royal Infirmary. She is well and normal at present (eighteen months later) on grs. 8 thyroid a day. So convinced was she of the principle of cause and effect that the child was christened Thyra to commemorate its origin!

*Sterility due to Pelvic Peritoneal Inflammation.*—This is, to my mind, the commonest of all the classes of sterility. The peritonitis may have originated in childhood and have been quite forgotten, a history of "inflammation of the bowels" being the only sign-post to go by. I have had many cases following operations for appendix abscess, and of course the usual ascending pelvic infections account for many. The "one-child marriage," so often due to mild puerperal infection (frequently gonococcal), may be classed with this group, though it is not, strictly speaking, sterility. It is in these cases that the greatest number of curettings and dilatings have been done with no effect.

The patient usually has some symptoms to show to account for her troubles. There is possibly a mild cervicitis, and very often a retroverted uterus. This is replaced, and she is then curetted, and as no pregnancy follows, the process is repeated with like results. The real trouble lies in the tubes and, possibly, the ovaries.

The great anatomical defect of the female organism is the presence of a passage between her peritoneal cavity and the surface of the body (vaginal canal), viz. the Fallopian tubes. These tend to be very easily obliterated, or kinked and obstructed, when any ascending infection takes place. In either case sterility is the result, as the ova and spermatozoa cannot come into contact with each other; even if they do, a tubal pregnancy may result. Furthermore, cases will often be found in which the whole of both ovaries are covered over by a "skin" of adhesions which may effectually prevent the escape of ova into the tube.

The frequency of a mild pelvic peritonitis will be granted by anyone who has had experience of abdominal surgery in women. It is almost the exception to find an abdomen in which there are no pelvic adhesions at all. And kinks and obliteration of both tubes may be present without any gross lesion which can be detected by ordinary methods of examination.

It is in these cases that the method of testing the patency of the tubes by passing carbon dioxide through them is so valuable, and I am much indebted to Major V. B. Green-Armytage, I.M.S., of the Eden Hospital, Calcutta, for drawing my attention to the ease and safety of the method about to be described.

Impey [1] wrote enthusiastically on this subject, and Victor Bonney has several times described its value, but the numerous papers by Provis amd Rubin have set this method of diagnosis on a firm basis.

The method is simple, and can be applied in the consulting room or bedroom without difficulty. The apparatus (Allen and Hanbury's Provis apparatus) consists of a "Sparklet" syphon adapted to fit to a wash bottle with a special glass delivery bell which lets out a bubble of 10 cc. of gas. This is connected to a catheter about 10 inches long made of flexible metal, with a cone-shaped obturator ½ inch from its extremity. Interposed is an aneroid manometer. The whole apparatus is carried in a box the size and shape of a microscope case and is quite light.

Two precautions only are needed: (*a*) the test must not be made within five days of a period, as the endometrial congestion may give a false reading; (*b*) there must be no profuse cervical discharge.

The method employed is to pass a speculum and then to mop the cervix over with *Tr. Iodi*, passing a little up the canal on the Playfair's probe. (Provis has had no peritonitis or salpingitis in 1,000 cases.) If necessary, a bullet forceps vulsellum is placed on the cervix, and the catheter is pushed into the cervical canal till the cone-shaped obturator is firmly pressed into the external os. The gas is now turned on, and it should pass over at a rate of roughly one bubble (=10 cc.) in twenty seconds. The manometer is then watched carefully. In a *normal* case gas passes into the peritoneal cavity at about 110—130 mm. Hg.

Not more than three bubbles should be passed in. It is easy to detect the pneumoperitoneum, and the gas can be heard to pass in if listened for with a stethoscope. Any escape into the vagina is at once audible, and it is quite unnecessary to fill that canal with fluid as has been suggested. The patient can be tested with equal ease on the back or in the lateral position. Personally I use the latter, as it is less unusual for the patient.

In an *abnormal* case one of two conditions may be found : (*a*) complete block of both tubes, or (*b*) " sticky" tubes, kinked or partially obliterated.

In the case of complete occlusion, the manometer will rise steadily to 200 mm. Hg., beyond which it is not safe to go, and the patient usually experiences some pain at this stage. The sterility can now be diagnosed with certainty as due to occlusion of the tubes, and the question of abdominal section can be discussed as the only possible—and very dubious—remedy.

In a number of cases the pressure rises to 160 mm. and then suddenly falls to 120, gas passes into the peritoneal cavity, and the patency of one tube at any rate is restored. It may be possible to feel an elastic swelling on one side, which is the still obstructed tube partly distended with gas. This procedure is really curative in certain cases. A relative of mine consulted Professor Blair Bell for sterility following one parturition. Gas was passed at 170 mm. and again ten days later at 130. She became pregnant within fourteen days, though this had not occurred for four years previously. I have had a similar experience in my own practice, and one has occurred at the Royal Infirmary.

On the question of operation for complete obstruction of both tubes I am very dubious. I have now performed salpingostomy on over twenty occasions, and only one pregnancy has resulted ; and that in a case where there were only a few adhesions binding the tip of the tube to the appendix. I have found that, if no pain at all is caused by 200 mm. pressure, the tube is obliterated at the uterine end, and I regard this as almost hopeless. If, however, discomfort is caused, I think that it indicates that some portion of the lumen of one tube at least is patent, for the thin tube stretches and causes pain much more easily than the firm, thick-walled uterus. In two of my Infirmary patients—

who suffered no discomfort from the 200 mm. pressure —a most interesting condition was found, which appeared to be a congenital absence of the lumen of both tubes. There was a thin fibrous cord within the peritoneal covering, and the pathological report stated that no signs of a lumen were present. In these two cases, at their urgent request, Tuffier's operation, of implanting the ovary into the uterine cavity, was carried out; but I told them the results were very uncertain, and so far (twelve months) no pregnancy has resulted, though the patients have suffered no discomfort. In those cases, where the gas passes freely at 120 mm. and the uterus is "cochleate" and ill developed, I dilate up to Hegar 14, and five of these patients have conceived, one unfortunately developing an ovarian pregnancy.

### SUMMARY.

(1) The chief essential in the treatment of sterility is an accurate diagnosis of the cause, not forgetting the husband.

(2) Anatomical defects and gross pathological lesions having been excluded, the most likely causes are endocrine upset, and mild pelvic peritonitis causing obliteration or kinks in the tubes.

(3) Thyroid gland in *large* doses is efficient in the endocrine cases.

(4) A test of the patency of the tubes should always be made before operative procedures are advised; otherwise many cases will be submitted fruitlessly to dilating and similar operations, involving the patient in loss of time and money, and discrediting the operator.

### REFERENCE.

[1] Impey, *Trans. Edinburgh Obstet. Society*, 1921–1922.

